# Genetic basis and pathogenesis of Familial WPW Syndrome

**Published:** 2003-10-01

**Authors:** Jasvinder Sidhu, Robert Roberts

**Affiliations:** Section of Cardiology, Baylor College of Medicine, Houston, Texas

## Introduction

The Wolff-Parkinson-White (WPW) syndrome has been a known clinical entity for over fifty years. In 1967 Durrer et al postulated WPW syndrome was due to an accessory pathway, bypassing the AV node, from the atria to the ventricles [1]. This was later confirmed by epicardial mapping. WPW is the second most common cause of paroxysmal supraventricular tachycardias in the western world and the most common cause in China [2]. WPW syndrome has a prevalence of 1.5 to 3.1 per 1000 persons in western countries [3-5] . Patients with the WPW syndrome may present with palpitations, presyncope, syncope, or sudden cardiac death (SCD). In some patients the first and only manifestation of the disease is SCD. This is more likely to occur in the setting of atrial fibrillation with a rapid ventricular response.

Electrocardiographic findings of WPW syndrome consist of preexcitation manifested by a shortened PR interval (<120 msec), a widened QRS (>100 msec) a delta wave (abnormal initial QRS vector) and supraventricular tachycardia. The EKG finding of preexcitation is a result of early ventricular depolarization through the accessory pathway. SVT can occur with retrograde or antigrade conduction through the accessory pathway forming the basis of the so-called “macro-reentrant” arrhythmia model [6]. Rapid conduction through the accessory pathway in the setting of atrial fibrillation markedly increases the risk of SCD [7].

We recently identified the gene responsible for familial Wolff-Parkinson-White [8]. The gene (PRKAG2) which encodes for a protein AMPK (AMP- activated protein kinase) was identified as the causal gene. Missense (single nucleotide change) mutations in this gene were identified in families with WPW. Six such mutations have been identified. Along with preexcitation the affected families had conduction abnormalities (AV block) and cardiac hypertrophy.

## Genetics of WPW

Familial WPW syndrome has an autosomal dominant mode of inheritance. In autosomal dominant inheritance 50% of the offspring inherit the mutated gene and are at risk of developing the disease and affects males and females equally.

In autosomal dominant mode of inheritance only one copy of the gene has to be mutated to cause the disease as opposed to autosomal recessive where both copies must have the mutations. We first performed genetic linkage analysis to locate the chromosomal location (locus) followed by the candidate gene approach to identify the gene in a family with an inherited form of WPW. The locus was on chromosome 7 (7q3) and the gene identified to be PKRAG2 which encodes for the gamma-2 subunit (non-catalytic subunit) of AMPK. Since this initial discovery five more mutations in the same gene have been identified. All of the mutations have been missense mutations in the PKRAG2 gene. Missense mutations are mutations in which a single nucleotide is substituted by another nucleotide inducing a change of a single amino acid in the final protein product Table 1).

AMPK is a protein that is made up of three subunits an alpha subunit, beta subunit (38 kDa) and a gamma subunit (36 kDa). PKRAG2 is the gene that encodes for the gamma-2 subunit of AMPK and it contains 16 exons (the region of the gene that is translated into amino acids) and is made up of 569 amino acids.

## Functions of AMPK

AMPK is a heterotrimeric protein which consists of alpha, beta, and gamma subunits. The gamma subunit has been characterized as the non-catalytic subunit. There are at least three isoforms of the gamma subunit (PKRAG1, PKRAG2, and PKRAG3). PKRAG1 and PKRAG2 are highly expressed in skeletal and cardiac muscle, whereas PKRAG3 expression is restricted to skeletal muscle [9]. Familial WPW syndrome is caused by mutations in the gamma-2 isoform (PRKAG2) of AMPK.

AMP-activated protein kinase (AMPK) serves as an energy sensor for the cell [9]. In times of energy depletion and/or stress it is activated. Stressors that have been known to activate AMPK include hypoxia, ischemia, exercise, and starvation [10]. This enzyme is activated by high AMP/ATP ratios and deactivated by low AMP/ATP ratios [10]. In energy depleted states (low ATP, high AMP) it is phosphorylated by AMPKK and is transformed to its active form. This process is reversed when ATP levels are restored. ATP interferes with the AMP/AMPK interaction and diminishes the ability of AMPKK to phosphorylate AMPK. Therefore it is the ratio of AMP/ATP that determines the level of AMPK activation.

AMPK has a multitude of physiological roles. Primarily it increases fatty acid oxidation and glucose uptake during times of stress [10] and it also decreases glycogen synthesis. AMPK inhibits acetyl-coA carboxylase it leads to a decrease in the synthesis of malonyl CoA. Since malonyl CoA is an inhibitor of fatty acid oxidation this leads to increased fatty acid oxidation. AMPK increases GLUT-4 a protein responsible for transport of glucose from the extracellular space to the intracellular space, thereby making glucose available for ATP production. In vitro studies have demonstrated that activation of AMPK inhibits glycogen synthase thus decreasing the amount of intercellular glycogen. These actions of AMPK classify it as a catabolic enzyme that responds to stress to maintain energy homeostasis.

## Clinical Characteristics of WPW syndrome

WPW can present clinically with palpitations, pre-syncope, syncope or SCD (sudden cardiac death). Familial WPW syndrome was described in a large French-Canadian family in 1986 [11]. In this family the members that were affected showed clinical findings that consisted of preexcitation, conduction abnormalities, and cardiac hypertrophy. We were fortunate to obtain access to the family and identify the responsible gene.

Preexcitation is defined as early depolarization of the ventricles. This early depolarization is manifested on a 12-lead EKG, as a short PR interval (<120 msec), and prolonged QRS (>100 msec), with the appearance of a delta wave (slurring of the QRS). Adult cardiac conduction begins at the SA-node and proceeds through the atria, AV-node, HIS-bundle, left/right bundles, throughout the ventricles. Patients with familial WPW syndrome have an accessory pathway that bypasses the normal cardiac conduction. The accessory pathway is usually an accessory conduction bundle connecting the atria and the ventricles. Hence the normal delay that occurs at the AV-node can be avoided by conduction through the accessory pathway. The presence of an accessory pathway also provides a reentrant circuit and the risk of developing SVT (supraventricular tachycardias). The development of SVT in the presence of preexcitation constitutes the WPW syndrome [12].

Familial WPW syndrome differs from other forms of WPW syndrome in that affected individuals also develop cardiac conduction abnormalities usually around the fourth decade of life [8]. Conduction abnormalities involve AV-nodal, ventricular as well as the accessory pathway. Treatment of patients often requires placement of a permanent pacemaker. It is thought this slowing is secondary to diffuse progressive myopathic process [13].

Family members afflicted with familial WPW syndrome also develop thickening of the myocardium. Cardiac hypertrophy has been demonstrated in these patients by ultrasound. The development of cardiac hypertrophy secondary to pressure overload (chronic hypertension, aortic stenosis) and sarcomeric mutations (HCM) are due to increased cell growth [14]. The hypertrophy found in families with WPW is also attributed to increased cell growth. It has been suggested that deposition of a glycogen-like substance may contribute to cardiac hypertrophy.

## Pathogenesis of Familial WPW syndrome

Families with WPW syndrome exhibit a variable phenotype consisting of cardiac hypertrophy, preexcitation, and conduction abnormalities. The syndrome is caused by missense mutations (single nucleotide change) in the gene PKRAG2 which encodes for the gamma-2 subunit of AMPK. There has been an explosion of knowledge on AMPK within the last decade. Initially it was a surprise that a gene that encodes a metabolic protein was involved in the generation of cardiac abnormalities. However, given AMPK’s vast array of functions it is perhaps not so surprising. The cardiac syndrome could be the result of a derangement of one or more of AMPK’s functions.

Pathophysiologically the syndrome due to PKRAG2 resembles other glycogen storage diseases such as Pompe disease [15]. The triad of cardiac hypertrophy, preexcitation, and conduction abnormalities has been documented in these diseases[16,17]. Therefore, the initial focus on the PRKAG2 induced cardiac syndrome has been on glycogen storage.

Cardiac hypertrophy has been demonstrated by ultrasound in patients with familial WPW syndrome[17]. The accumulation of glycogen-like substance has also been seen in the myocardium of these patients[18]. The increased thickness of the myocardium (cardiac hypertrophy) has been attributed to the excessive deposition of glycogen-like substance as well as increased myocyte growth, which maybe somewhat different from the hypertrophy seen in HCM (hypertrophic cardiomyopathy) which is secondary only to increased myocyte growth and fibrosis.

Conduction abnormalities (heart block or slowing of the conduction system) can be explained by two well established facts. First glycogen is known to be more abundant in the conduction system compared to the myocardial muscle tissue [13]. Therefore excess accumulation of glycogen-like substance could lead to loss of conduction tissue and slowing or block. Secondly excessive glycogen has been known to be toxic to the conduction system which could lead to loss of function [13].

WPW syndrome in humans is caused by the existence of an accessory pathway. Initially there is no separation between the atria and the ventricles. During development either through apoptosis or remodeling the atria separates from the ventricle. It is possible that the normal developmental process of apoptosis and remodeling doesn’t occur in patients with familial WPW syndrome. Therefore some myocardial tissue that remains as a connection between the atrium and the ventricle [18,19].

AMPK’s role in the development of the pathophysiology in familial WPW could be a result of gain in function or a loss in function. The increased amount of glycogen found in the myocardium of these patients suggests a loss of function if we believe that AMPK activity decreases glycogen stores. Hypertrophy would be explainable since a loss of function would lead to an ATP deficient state and increased cell growth as compensation for increased work load. Conduction abnormalities could arise from the deposition of glycogen-like substance in the conduction system. Preexcitation maybe a result of a developmental abnormality as explained above.

## Animal model of Familial WPW syndrome

Transgenic technology has been used in biomedical research over the last 20 years. The basic idea is to introduce a gene with a known phenotype into an animal in the hope the offspring will manifest the phenotype. Since the mutation for WPW syndrome was identified interest in generating a transgenic model for the disease has intensified. Recently a mouse model of this human disease has been developed (Seidman et al) [20]. The generation of a transgenic model usually is done by cloning the mutated gene of interest along side a powerful promoter in the expectation that the gene will be overexpressed. In Seidman’s model the gene of interest was the mutated PRKAG2 gene which encodes for the gamma2 subunit of AMPK and the promoter used was the alpha-myosin heavy chain promoter.

The mice generated recapitulated the human phenotype with preexcitation and cardiac hypertrophy. Histopathology demonstrated vacuoles that appear to be like those seen in the human phenotype. Interestingly Seidman et al demonstrated increased AMPK activity in the mutant transgenic mice versus the wild type transgenic mice. This would suggest that the missense mutation leads to a gain in function of AMPK. Electrophysiological studies done on these mice demonstrated the existence of an accessory pathway consistent with the human form of WPW. Seidman et al proposes that the glycogen deposition disrupts the annulus fibrosa which is responsible for preexcitation.

We recently developed a transgenic animal (mice) model for human familial WPW syndrome. Our model has a phenotype of preexcitation and evidence for an accessory pathway. In our model we were able to induce SVT.

## Future Considerations

Although WPW in its classical form is usually treated with catheter ablation and conduction abnormalities (AV block or slowing of conduction) can be handled clinically with the placement of a permanent pacemaker. There is definitely a role for genetic screening in the setting of familial WPW syndrome. Screening will allow the physician to identify individuals and families at risk for developing AV block and cardiac hypertrophy. This may become even more important in the future if novel treatments can be found for this glycogen storage disease.

The development of animal models will also give insight into the molecular mechanisms involved in the pathogenesis of this disease. This will also help understand normal development of the cardiac conduction system.

## Figures and Tables

**Table 1:**
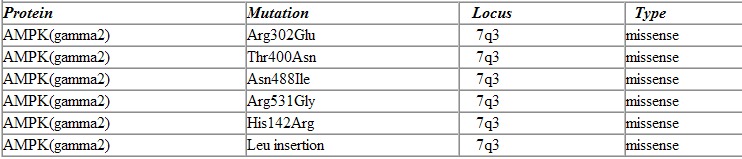
Mutations in PRKAG2 responsible for Human WPW syndrome
